# Development of a Theory-Based Intervention to Enhance Information Exchange during Over-The-Counter Consultations in Community Pharmacy

**DOI:** 10.3390/pharmacy6040117

**Published:** 2018-10-24

**Authors:** Liza J. Seubert, Kerry Whitelaw, Laetitia Hattingh, Margaret C. Watson, Rhonda M. Clifford

**Affiliations:** 1 Division of Pharmacy, The University of Western Australia, M315, 35 Stirling Highway, Crawley, WA 6009, Australia; kerry.whitelaw@uwa.edu.au (K.W.); rhonda.clifford@uwa.edu.au (R.M.C.); 2 School of Pharmacy and Pharmacology, Griffith University, Gold Coast Campus, Queensland 4222, Australia; l.hattingh@griffith.edu.au; 3 Department of Pharmacy and Pharmacology, University of Bath, 5W 3.33, Claverton Down, Bath BA2 7AY, UK; m.c.watson@bath.ac.uk

**Keywords:** communication, nonprescription drugs, pharmacists, community pharmacy services, behaviour change, health behaviour

## Abstract

(1) Background: Community pharmacy personnel help mitigate risks of self-care by consumers who seek over-the-counter (OTC) medicines or treatment of symptoms and/or conditions. Exchange of information facilitates the OTC consultation, but pharmacy personnel often report difficulties in engaging consumers in a dialogue. The aim of this study was to describe the development of a behaviour change intervention to enhance information exchange between pharmacy personnel and consumers during OTC consultations in community pharmacies. (2) Methods: The Behaviour Change Wheel methodological framework was used to link factors that influence consumer engagement with information exchange during OTC consultations with intervention functions to change behaviour. Options generated were rationalized and the final intervention strategy was derived. (3) Results: Education, persuasion, environmental restructuring, and modelling were determined to be potential intervention functions. The intervention incorporated placing situational cues in the form of posters in the community pharmacy modelling information exchange behaviour, persuading through highlighting the benefits of exchanging information and educating about its importance. (4) Conclusions: A systematic, theoretically underpinned approach was applied to develop candidate interventions to promote information exchange in OTC consultations. The feasibility and efficacy of the intervention strategy has since been tested and will be reported elsewhere.

## 1. Introduction

Community pharmacy personnel manage over-the-counter (OTC) enquiries every day [1,2], which include requests for named OTC medicines as well as the treatment of symptoms and/or conditions [3,4]. Consumers are becoming increasingly confident in self-managing minor ailments by using information from a variety of sources, such as the internet, to self-diagnose and select medicines they view to be appropriate [5,6]. This is facilitated by the wide range of OTC medicines available from community pharmacies in many countries, which require varying levels of involvement by pharmacy personnel, depending on the legal classification and regulation [7,8,9]. Furthermore, community pharmacies are accessible, often with extended opening hours, and without the need to book an appointment to see a pharmacist [10,11,12,13]. 

Benefits of consumers in engaging with self-care for minor ailments include convenience, and time and cost savings [6,14]. However, there is a risk that consumers could misdiagnose their condition(s), resulting in delays in initiating appropriate treatment [6,14]. Consumers may also underestimate the risks of OTC medicines, which could result in adverse effects [15,16,17,18,19]. Pharmacy personnel play an important role in mitigating the risks associated with self-care. Community pharmacists are qualified to manage the complexity of OTC enquiries in the community pharmacy setting by engaging with consumers in a consultation [20,21,22]. Gathering information from consumers about the symptom or condition, the person’s medical history and medicines, and their treatment goals, assists pharmacists in providing appropriate recommendations [2,23]. Many factors influence information exchange during OTC consultations, including the communication skills of pharmacy personnel, consumer expectation to purchase an OTC medicine without needing to answer questions, privacy, and the legal classification of the medicine [24,25,26,27,28,29,30,31,32,33,34,35,36,37]. 

Pharmacists and pharmacy personnel often report difficulties in engaging consumers in a dialogue, particularly when the request is for a specific medicine by name [25,27,38,39,40]. OTC consultations ideally should involve two-way communication “between the pharmacist and the patient in which the pharmacist ascertains the needs of the patient and provides them with information required to effectively use medicines and/or therapeutic devices” [20] (p. 50). This interaction requires clinical knowledge and reasoning, as well as effective communication. There is substantial evidence, however, that the management of the diverse range of OTC enquiries encountered in community pharmacies is sub-optimal, and that this is mainly due to inadequate information gathering and/or advice or information provision by pharmacy personnel [1,26,27,28,29,31,32,41,42,43,44,45,46]. While there has been a number of interventions to improve the exchange of information between pharmacy personnel and consumers, with varying levels of success [47], there are also studies which show that pharmacy personnel are not complying with appropriate standards [26,29,41,42,43,45].

The aim of this study was to describe the development of a behaviour change intervention to enhance information exchange between pharmacy personnel and consumers during OTC enquiries in community pharmacies.

## 2. Materials and Methods

**Figure 1:**
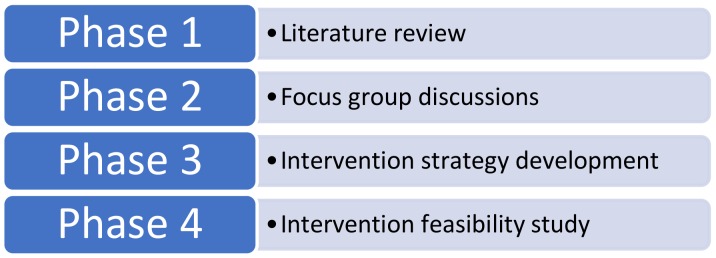
Project phases.

This study was the third phase of a larger project with the aim of enhancing the quality management of OTC consultations in community pharmacies (Figure 1). 

In the first phase, a systematic literature review identified interventions targeted towards improving communication between consumers and pharmacy personnel during OTC consultations in the community pharmacy setting [47]. Focus group discussions were then conducted to determine pharmacist, non-pharmacist pharmacy personnel and consumer perspectives regarding barriers and facilitators for information exchange during OTC consultations in community pharmacies [39]. The results from the first two phases identified that to enhance information exchange between pharmacy personnel and consumers during OTC enquiries, consumers needed to engage with the process. The methodology described in the Behaviour Change Wheel (BCW) [48] was subsequently used to develop an intervention strategy to target this behaviour.

### Underpinning Theory

The BCW is a validated methodological framework developed from the synthesis of 19 behaviour change frameworks to assist researchers to apply the COM-B (Capability Opportunity Motivation—Behaviour) model of behaviour in any setting to develop an intervention strategy. The BCW identifies sources of behaviour in terms of the complex interactions between capability, opportunity and motivation.

The Theoretical Domains Framework (TDF) [49] was also applied in this study. The TDF is a validated derivation of the COM-B which identifies 14 domains that determine behaviour. In Table 1, the TDF domains are linked to source behaviours of COM-B. An analysis using TDF provides a more detailed understanding of determinants of behaviour from which an intervention strategy can be developed.

The BCW describes interventions in terms of nine functions: (i) education, (ii) persuasion, (iii) incentivisation, (iv) coercion, (v) training, (vi) restriction, (vii) environmental restructuring, (viii) modelling and (ix) enablement. A function of an intervention is an aspect of the intervention that influences behaviour. The BCW links intervention functions with behaviour change techniques (BCTs), which are the active components that can be used in the intervention strategy [50]. The BCTs are assessed against the APEASE (Affordability, Practicability, Effectiveness and cost effectiveness, Acceptability, Side-effects/safety, Equity) [51] criteria and to enable decisions on intervention content and delivery that are within the scope of the study.

In Phase 3, a 2-stage process was used to develop the intervention. In Stage 1, to fully understand the target behaviour, an independent duplicate (LS, KW) behavioural ‘diagnosis’ of consumer engagement with information exchange during OTC consultations was conducted, using themes from the focus groups undertaken in Phase 2 [39]. The researchers coded focus group themes to the COM-B model and TDF. The results were discussed with a psychologist experienced in pharmacy practice (L. Smith, see Acknowledgments) until consensus was reached. Independent duplicate (LS, KW) mapping of the key factors that influenced this behaviour to intervention functions and BCTs [48] was conducted (Stage 2). Disagreements were resolved by consensus and involvement of a third researcher, when required (RC). Options for the intervention were generated by the research team (LS, KW, LH, MW, RC) then rationalised (LS, KW) through assessment against the APEASE criteria [51] and discussion. The final intervention strategy was decided by consensus (LS, KW, LH, MW, RC).

## 3. Results

### 3.1. Stage 1: Behavioural Analysis

A behavioural diagnosis on the target behaviour, as described in the BCW and resulting COM-B and TDF coded themes from Phase 2 focus group discussions, was conducted (Table 2).

### 3.2. Stage 2: Identify Intervention Options, Content and Implementation Options

Education, persuasion, environmental restructuring, and modelling were determined to be potential intervention functions (Table 3) that met the APEASE criteria. 

The BCTs identified to be able to deliver the four intervention functions are listed in Table 3 with examples of BCTs to address identified barriers.

### 3.3. Intervention Strategy

The research team discussed the results of the analysis and developed the intervention. Situational cues, in the form of a poster displayed in a community pharmacy (**environmental restructuring**), depicting consumers with OTC enquiries engaging in information exchange (**modelling**), highlighting the benefit of this behaviour (**persuasion**) and the reasons why it is important (**education**), were identified as the most appropriate intervention. A second poster depicting a pharmacist and information about the qualifications and role of a pharmacist was developed. An additional situational cue, in the form of a badge, was developed to be worn by pharmacy personnel to identify their position as either pharmacist or pharmacy assistant. 

## 4. Discussion

This study described the development of an intervention strategy to enhance information exchange during OTC consultations. With the growing trend for self-care and the empowerment of consumers to make health decisions, it is essential that their safety is protected through the expertise available from pharmacists and other pharmacy personnel. 

A systematic, theory-based approach was undertaken to fully understand the key components impacting information exchange. 

The target of most interventions to date has been pharmacy personnel, with varying degrees of success [47]. Interventions targeting consumers have been neglected [47]. Through the systematic process, the behaviour of interest was identified to be information exchange and the consumer’s engagement in information exchange was determined to be the target as there was a scarcity of interventions directed at the consumer. 

The scope of this study was to derive an intervention to promote information exchange. The strength of this study lies in using a rigorously developed theory-based methodology for the systematic development of an intervention. The steps involved in the intervention development are described in detail, therefore making it reproducible. 

## 5. Conclusions

A systematic, theoretically underpinned approach was applied to candidate interventions to promote information exchange in OTC consultations. The feasibility and efficacy of the intervention has since been tested and will be reported elsewhere.

## Figures and Tables

**Table 1:** Capability, opportunity, motivation–behaviour model (COM-B) linked with the Theoretical Domains Framework (TDF) domains.

COM-B Source Behaviour	TDF Domain
CAPABILITY	Skills (Cognitive and interpersonal; Physical)
	Knowledge
	Memory, attention and decision processes
	Behavioural regulation
OPPORTUNITY	Social influences
	Environmental context and resources
MOTIVATION	Social and professional role and identity
	Belief about capabilities
	Optimism
	Belief about consequences
	Intentions
	Goals
	Reinforcement
	Emotion

**Table 2:** Behavioural diagnosis using themes from focus group meetings.

Target Behaviour: Consumer Engaging in Information Exchange.			
COM-B and TDF *	Barrier	Is There a Need for Change?	Intervention Function
**PSYCHOLOGICAL CAPABILITY**			
**Knowledge** (An awareness of the existence of something) [52]	Consumers did not understand the role and responsibilities of pharmacists.	√	Education
	Consumers did not understand the qualifications of pharmacists.	√	
	Consumers did not understand the risks of medicine use.	√ Consumers do not perceive risks with OTC medicines. Consumers believe medicines available without prescription are safe.	
**Cognitive and interpersonal skills** (An ability or proficiency acquired through practice) [52]	Pharmacy personnel consultation & communication skills	Improving these skills may improve interactions.	Training
**PHYSICAL OPPORTUNITY**			
**Environmental context and resources** (Any circumstance of a person’s situation or environment that discourages or encourages the development of skills and abilities, independence, social competence, and adaptive behaviour) [52]	Privacy is required for conversations.	√ Discussing health can be a sensitive issue.	Training Restriction Environmental restructuring Enablement
	Pharmacy personnel should have time to engage in interactions	√	
	Pharmacists were not always identifiable	√	
	Appropriate remuneration for pharmacist consultations is required	√	
	The environment should look like a professional/healthcare setting	Potentially yes. Some community pharmacies are very retail/warehouse/discount oriented.	
	The OTC consultation area is not always clearly identifiable	√	
**REFLECTIVE MOTIVATION**			
**Social and professional role and identity** (A coherent set of behaviours and displayed personal qualities of an individual in a social or work setting) [52]	Consumers did not trust the person asking questions	√ Consumers do not know the role of the pharmacist	Education Persuasion Modelling
	Service between pharmacies and personnel is not consistent so consumers did not know what to expect	√	
**Belief about capabilities** (Acceptance of the truth, reality, or validity about an ability, talent, or facility that a person can put to constructive use) [52]	Consumers believed they are able to appropriately self-asses their condition before consultation	√	Education Persuasion Modelling Enablement
	Consumers did not believe pharmacy personnel were able to help with OTC enquiries	√	
**Belief about consequences** (Acceptance of the truth, reality, or validity regarding outcomes of a behaviour in a given situation) [52]	Consumers did not understand the risks of medicine use	√ Consumers engage in information exchange if they ask about a symptom but not if they ask for a specific product	Education Persuasion Modelling
	Consumers did not know that being asked questions is for their benefit	√	
	Consumers did not know that their consultation information will be kept confidential	√ Consumers do not know that pharmacy personnel are bound by privacy laws	
**Intentions** (A conscious decision to perform a behaviour or a resolve to act in a certain way) [52]	Consumers expected to purchase an OTC product without exchanging information	√	Education Persuasion Incentivisation Coercion Modelling
	Consumers expected to answer questions if asking about a symptom	√	
	Consumers resisted information exchange if repeatedly requesting the same product	√	
**AUTOMATIC MOTIVATION**			
**Reinforcement** (Increasing the probability of a response by arranging a dependent relationship, or contingency, between the response and a given stimulus) [52]	Consumers did not feel it necessary to be asked questions (not from focus group but an observation of the research group)	√ If consumers exchange information and have a positive outcome as a result, this will subconsciously encourage information exchange behaviours in future consultations.	Training Incentivisation Coercion Environmental restructuring
**Behavioural diagnosis of the relevant COM-B components**	**Psychological capability, physical opportunity, reflective and automatic motivation need to change in order for the target behaviour “consumer engaging in information exchange” to occur.**		

* COM-B: Capability Opportunity Motivation—Behaviour model of behaviour; TDF: Theoretical Domains Framework.

**Table 3:** Linking intervention functions to Behaviour Change Techniques (BCTs).

Intervention Function	BCTs Identified to Enable Delivery of the Intervention Function	BCT Examples
Education	Information about social and environmental consequences Information about health consequences Prompts/cues	Explain the role and responsibilities of the pharmacist. Explain the qualifications of the pharmacist. Explain the risks of OTC medicine use. Explain the confidentiality of personal information.
Persuasion	Credible source Information about health consequences	Inform consumers about positive health consequences from information exchange.
Environmental restructuring	Adding objects to the environment Prompts/cues	Pharmacy personnel to wear badges identifying their role. Provide cues/prompts for engaging in information exchange.
Modelling	Demonstration of the behaviour	Demonstrate the type of questions that might be asked.
